# Supplementary figures and images for: COVID-19 vaccine hesitancy among the adult population in Bangladesh: A nationwide cross-sectional survey
Source: PLoS One. 2021 Dec 9;16(12):e0260821. doi: 10.1371/journal.pone.0260821 (PMC8659424; doi:10.1371/journal.pone.0260821)

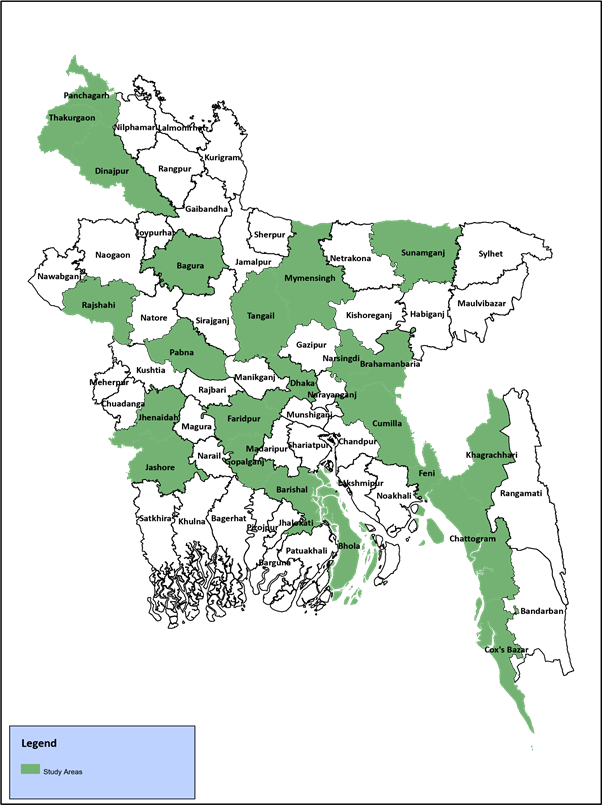

Supplement: S1 Map — (TIF) [file pone.0260821.s001.tif]
