# Supplementary material for: COVID-19 vaccine hesitancy among the adult population in Bangladesh: A nationwide cross-sectional survey
Source: PLoS One. 2021 Dec 9;16(12):e0260821. doi: 10.1371/journal.pone.0260821 (PMC8659424; doi:10.1371/journal.pone.0260821)
Supplement: Attachment — Submitted filename: Response to reviewers.docx [file pone.0260821.s002.docx]

**PONE-D-21-14374
COVID-19 Vaccine Hesitancy among the Adult Population in Bangladesh: A Nationwide Cross-sectional Survey**

| **Serial** | **Observations** | **Comments on Observations** |
| --- | --- | --- |
| **Reviewer 1** | | |
| 01 | Please mention how the items for the various scales used in the study were developed and validated, | Thanks for your attention on this important point. We have addressed this issue in the lines between 163-173 of the cleaned version, which reads as follows:  ***We selected variables and items for constructing scales from the previous studies [12,28–32] and then mixed and customized the different items to develop the scale for the Bangladesh context. The data collection tool was pretested to validate using the face-to-face interview to determine respondents' understanding of the questions, comprehensiveness of the questionnaire, as well as wording, length and sensitivity of the questions. We calculated internal consistency using Cronbach’s alpha (α) to assess the reliability of the items used in the scales. We developed a total of ten scales, of which seven scales had an α between 0.700 to 0.857, and three scales had an α between 0.612 to 0.657. The reliability analysis of seventy percent of our scales was good as the α ranged between 0.7 and 0.8 [33]. The discussion below provides a detailed discussion on scale development, and the items used in these scales are available at*** [***https://osf.io/e4xph/***](https://osf.io/e4xph/)***.*** |
| 02 | Mean score of the hesitancy scale should not be used to calculate hesitancy rather the proportion of participants who were not willing to take/recommend the vaccine to family members should be considered for vaccine hesitancy. Use of the previous method has led to overestimation of vaccine hesitancy in the study. | Thanks for this comment. We agree with you and have followed your recommendation and revised the calculation, reflected in the lines between 176-190 of the cleaned version. However, the new calculation increased the hesitancy rate. Now the hesitancy rate is 46.2% which was 41.1% earlier. |
| 03 | Accuracy of the scale cannot be calculated by dividing the mean score of the scale by overall score (Line 257-259). | Thanks for this comment, which is also related to the previous comment. We have deleted this line and followed your recommendation as mentioned above. |
| 04 | Comparing the proportion with vaccine hesitancy with the respondent’s characteristics will be more appropriate than mean score of vaccine hesitancy (Table 2). Computing the overall score for each predictor variable and categorising it and comparing it with the proportion with vaccine hesitancy would be more meaningful than comparing mean scores with each question under a predictor variable. Also, the responses of for each question under a predictor variable can be clubbed into categories namely agree, disagree and no opinion and then be compared (Table 3 and Table 4). | Thank you for this comment. We have followed you and revised the Table 2, 3, and 4. Accordingly, we have now used logistic regression instead of linear regression. Table 5 shows the findings of logistic regression. |
| **Reviewer 2** | | |
| 01 | Abstract - Abstract provides a balanced summary. The methods section under the abstract shall indicate the study setting (total administrative divisions included) and the duration of the study. | Thanks for this comment. We have added this in the abstract. Please see line 31 in the cleaned version. |
| 02 | Methods: - Describe study setting: total population of Bangladesh, proportion of urban or rural population, sex ratio, life expectancy, literacy rate, economic status, and other factors that have influence on the vaccine hesitancy. | We appreciate your comment to improve our manuscript by adding this point. We have described the study setting. Please see lines 108 to 119 of the cleaned version. |
|  | Please indicate the exact number of participants from the online and face-to-face surveys. | Thanks a lot for this important comment. We have added this information. Please see lines 130 to 131 of the cleaned version. |
|  | The sampling strategy used for selecting participants from the districts of administrative divisions of Bangladesh for the face-to-face interviews shall be included. Describe how (sampling procedure) and where (setting) the participants were chosen within each district. | Thanks a lot for highlighting this point. Please see lines 141 to 160 of the cleaned version where we have addressed your comment. |
|  | Please include the reference number and the date of approval of ethics clearance under the ‘ethical approval’ section | Please see line 249 of the cleaned version where we have addressed this query. |
|  | What was the rationale behind choosing margin of error of 0.03 and design effect of 1.6 in sample size calculation? | Thanks a lot for this important question. We have used the prevalence of vaccine hesitancy (p) from a previous study conducted in Bangladesh. The prevalence rate was 32.5% that means the value of p in our formula was 0.325. With this value of p, we could use a margin of error of 0.05, which is normally suggested. However, to increase our sample size considering a nationwide survey, we reduced the margin of error to increase the sample size. On the other hand, the design effect is normally suggested between 1.5 to 3.0. Thus, we adopted 1.6 appropriate for this study. |
|  | Please include what was considered as non-response by an individual during face-to-face interviews? | Thanks for this important question. The respondents who did not consent to take part in the study and who consented to participate in the study but did not know about the COVID-19 vaccine were considered as non-response. Please see lines 126 to 130 of the cleaned version where we have addressed this issue. |
|  | Regarding staff involved in face to face interviews: Who were involved in data collection and their training before data collection shall be described in brief. | Please see lines 157 to 160 of the cleaned version where we have described this. |
|  | The study claims to be a nationally representative survey. However, the methodology described doesn’t reflect a robust sampling strategy to support the claim. The limitations of the study admits the non-probability sampling method used and cautions the generalizability of the study findings. So, kindly justify or changes shall be made as appropriate in the manuscript. | Thanks a lot for your significant observation. We agree with you and thus, revised our title. |
|  | Results: - The results are described in detail and are in line with the proposed study objectives. However, I would like to see 95% CI for the summary estimates. | The findings of the adjusted odds ratio have been reported with 95%CI. Please see Table 5. |
|  | - Multiple sub-group analysis was done in this study and I am not sure if the sample size is enough to test for multiple statistical tests. Authors are requested to consult a bio-statisticians for analysis. | Thanks for your observation. We did not do any sub-group analysis, e.g., different analyses for men and women. We just conducted statistical analysis to establish the association between the outcome variable and independent variables. The analysis was used to select variables for multiple logistic regression. Our team has a Biostatistician, and we are confident that we had enough samples for conducting multiple regression analysis. |
|  | What were the response rates for the online and face-to-face surveys? Kindly include. | Thanks for this observation. Please see lines 126 to 130 of the cleaned version where we have addressed this issue. |
|  | - Please add (N=?) in the titles of the tables | We have addressed this comment in all the figures and tables. We appreciate your observation. |
|  | Discussion: - Region specific data (Bangladesh/other LMICs) on determinants of COVID-19 vaccine hesitancy shall be highlighted in the discussion. This could substantiate the study findings as well as broaden the scope of study results in the international context. | Thanks for this advice. Region specific data (Bangladesh/other LMICs) on determinants of COVID-19 vaccine hesitancy is highlighted now in the discussion. |
|  | It may be necessary to broaden to the public health dimension i.e. recommendations or possible actions that could be undertaken to deal with the determinants of COVID-19 vaccine hesitancy | Thanks for this significant comment. We have addressed this issue in the conclusion section now. |
