# Supplementary material for: COVID-19 vaccine hesitancy among the adult population in Bangladesh: A nationwide cross-sectional survey
Source: PLoS One. 2021 Dec 9;16(12):e0260821. doi: 10.1371/journal.pone.0260821 (PMC8659424; doi:10.1371/journal.pone.0260821)
Supplement: Attachment — Submitted filename: Response to reviewers Plain Text.docx [file pone.0260821.s003.docx]

**PONE-D-21-14374R1: COVID-19 Vaccine Hesitancy among the Adult Population in Bangladesh: A Nationwide Cross-sectional Survey**

**Reviewer 1**

**Comment 1:** Thank you for accepting and modifying the manuscript as per the suggestions. There's a lot of improvement in the manuscript. Though the manuscript has been written in an intelligible way, the language needs corrections at multiple places. The writeup appears to be verbose, can be pruned to convey the same message in lesser words.

**Our Response:** Thanks a lot for your comments. We have edited the manuscripts according to your suggestion.

**Reviewer 4:**

**Comment 1:** Sentences need to be framed in correct English, to be understandable by the reader. Line 62-63, Line 94 (should be plural-studies, as we aim to look for multiple related studies to say this fact) not very clear.

**Our Response:** Thanks a lot, pointing this out. We have taken care of this issue in the manuscript.

**Comment 2:** Table formatting and alignment needed overall.

**Our Response:** We are sorry that we could not identify any table formatting and alignment issue. We understand that it can be addressed during the production phase if there is any such issue.

**Comment 3:** Result section cannot have terms like about, nearly, near to. Be confident about the percentages obtained from your research.

**Our Response:** Thanks a lot for raising this critical point. We are confident about the percentages that we have reported in the table. We wrote in that style to avoid numbers at the beginning of the sentence. However, we have revised the manuscript thoroughly to address your comment.

**Comment 4:** Avoid using mass people repeatedly, mass in itself explains it (Line 83-84).

**Our Response:** Thank you. We have addressed this.

**Comment 5:** How was sample size decided for online and face to face interview? Needs more clarity on this.

**Our Response:** We decided to follow a 2:1 ratio for interviewing samples using face-to-face and the online survey considering the country's digital divide. We have provided this justification.

**Comment 6:** How were participants consented and approached for both the modes of data collection?

**Our Response:** Thanks a lot for raising this question. We have added a description of this issue in the Ethical Approval Participant’s Consent section.

**Comment 7:** Please specify inclusion and exclusion criteria clearly in the beginning of the study methodology. Study methodology needs more specification regarding study setting-mention names of the blocks in which study was set up. Presently everything is scattered over the places. It will create confusion in readers mind. Have proper sub headings for methodology section.

**Our Response:** We have thoroughly worked on these issues and revised our methodology section.

**Comment 8:** Fig 3, 4 , Table 3,4 needs percentage inclusion in paragraph written for them, Plain text not making much sense.

**Our Response:** Thanks a lot for this suggestion. We have revised figures 2 to 5 and tables 3 and 4.

**Comment 9:** Please justify why was non probability sampling was chosen.

**Our Response:** Thanks a lot for this question. Now, we have justified the adoption of non-probability sampling.
