# Supplementary material for: COVID-19 vaccine hesitancy among the adult population in Bangladesh: A nationwide cross-sectional survey
Source: PLoS One. 2021 Dec 9;16(12):e0260821. doi: 10.1371/journal.pone.0260821 (PMC8659424; doi:10.1371/journal.pone.0260821)
Supplement: Attachment — Submitted filename: Response to reviewers Plain Text.docx [file pone.0260821.s004.docx]

**PONE-D-21-14374R2: COVID-19 Vaccine Hesitancy among the Adult Population in Bangladesh: A Nationwide Cross-sectional Survey**

**Reviewer 4:**

**Comment-1:** Line 177-it should be respondents, remove single quotation mark.

**Our Response:** Thanks a lot for identifying this typo. We have removed the single quote.

**Comment 2:** Line 178-179, 181,183- double quotation marks instead of single are recommended.

**Our Response:** We appreciate your observation and we have modified accordingly.

**Comment 3:** Line 203-204, 209, single quotation can be removes, as in other places in the same paragraph. Please check for similar changes across the manuscript if present.

**Our Response:** We accept your observations and revised the manuscript accordingly. Thanks a lot.

**Comment 4:** Table -1, "Total" in the end is not well clear. In my opinion it can be removed, as table heading has n=1497 mentioned already.

**Our Response:** We agree with you and deleted the total.

**Comment 5:** Table 2 ,3 and 4, in the table title "%", can be removed, as the table below shows data well in % as mentioned in the column 2 of the table. Again, "Total ", not required in the end of the table in my opinion.

**Our Response:** Thanks a lot for your comments. We agree about the deletion of % from the table title of tables 2, 3, and 4. However, we believe the “Total” in table 2 is required as the reader will not be able to get this information from the rest of the table.

**Comment 6:** Line 364-365, "I think the complications.....Coronavirus", should be in quotations.

**Our Response:** Thanks a lot! We have used the double quote mark.

**Comment 7:** Footnote of the table 4 can be without quotation.

**Our Response:**  Thanks a lot! We have removed the quotation from the Footote.

**Comment 8:** Line 88 mentions HBM, need not repeat the full form in Line 246.

**Our Response:** Thanks a lot! We have corrected this.
